# Development of a novel instrument to characterize telemedicine programs in primary care

**DOI:** 10.1186/s12913-023-10130-5

**Published:** 2023-11-17

**Authors:** Logan D. Cho, Grace Rabinowitz, Crispin Goytia, Katerina Andreadis, Hsin - Hui Huang, Natalie C. Benda, Jenny J. Lin, Carol Horowitz, Rainu Kaushal, Jessica S. Ancker, Jashvant Poeran

**Affiliations:** 1 Icahn School of Medicine at Mount Sinai, 1 Gustave L. Levy Pl, New York, NY 10029 USA; 2 Department of Population Health Science & Policy, Icahn School of Medicine at Mount Sinai, 1425 Madison Avenue Box 1077, New York, NY 10029 USA; 3 Department of Population Health Sciences, Weill Cornell Medical College, 402 E. 67Th Street, New York, NY 10065 USA; 4 Institute for Healthcare Delivery Science, Department of Population Health Science & Policy, Icahn School of Medicine at Mount Sinai, 1425 Madison Avenue Box 1077, New York, NY 10029 USA; 5 Department of Orthopedics, Icahn School of Medicine at Mount Sinai, 1425 Madison Avenue Box 1077, New York, NY 10029 USA; 6 Department of Medicine, Division of General Internal Medicine, Icahn School of Medicine at Mount Sinai, 17 E. 102nd Street Box 1087, New York, NY 10029 USA; 7 Department of Biomedical Informatics, Vanderbilt University Medical Center, 2525 West End Ave., Rm 14122, Nashville, TN USA; 8 Department of Medicine, Icahn School of Medicine at Mount Sinai, 1425 Madison Avenue Box 1077, New York, NY 10029 USA

**Keywords:** Telemedicine, Primary care, COVID - 19 pandemic, Survey development

## Abstract

**Background:**

Given the rapid deployment of telemedicine at the onset of the COVID - 19 pandemic, updated assessment methods are needed to study and characterize telemedicine programs. We developed a novel semi - structured survey instrument to systematically describe the characteristics and implementation processes of telemedicine programs in primary care.

**Methods:**

In the context of a larger study aiming to describe telemedicine programs in primary care, a survey was developed in 3 iterative steps: 1) literature review to obtain a list of telemedicine features, facilitators, and barriers; 2) application of three evaluation frameworks; and 3) stakeholder engagement through a 2-stage feedback process. During survey refinement, items were tested against the evaluation frameworks while ensuring it could be completed within 20–25 min. Data reduction techniques were applied to explore opportunity for condensed variables/items.

**Results:**

Sixty initially identified telemedicine features were reduced to 32 items / questions after stakeholder feedback. Per the life cycle framework, respondents are asked to report a month in which their telemedicine program reached a steady state, i.e., “maturation”. Subsequent questions on telemedicine features are then stratified by telemedicine services offered at the pandemic onset and the reported point of maturation. Several open - ended questions allow for additional telemedicine experiences to be captured. Data reduction techniques revealed no indication for data reduction.

**Conclusion:**

This 32-item semi-structured survey standardizes the description of primary care telemedicine programs in terms of features as well as maturation process. This tool will facilitate evaluation of and comparisons between telemedicine programs across the United States, particularly those that were deployed at the pandemic onset.

**Supplementary Information:**

The online version contains supplementary material available at 10.1186/s12913-023-10130-5.

## Background

The onset of the COVID - 19 pandemic accelerated a shift towards virtual care in the United States, particularly in primary care settings, with telemedicine now being routinely offered to patients [1, 2]. Despite widespread availability of telemedicine, challenges remain as telemedicine programs were swiftly deployed, often in immature states, thus forgoing a traditional health information technology (IT) life cycle [3, 4]. The latter is of particular importance as evaluation throughout the entire health IT life cycle is thought to be crucial to ensure that any health IT intervention has time to reach its full potential [5, 6]. Such life cycle phases generally include the following: 1) planning, 2) design and development, 3) implementation and use, and 4) a fully operational phase with monitoring, evaluation, and optimization [3, 4].

The rapid implementation of telemedicine programs in primary care has complicated systematic evaluations with likely substantial variations in experiences between individual practices; for example, acuteness of telemedicine roll-out, available telemedicine features or resources may differ significantly between practices. Indeed, since the deployment of these telemedicine programs, some practices and providers have felt the strain of using hastily-developed, insufficiently mature programs that had not fully considered workflow and human factors [7]. Since the onset of the pandemic, several frameworks to evaluate telemedicine programs have been proposed. However, frameworks have focused on telemedicine quality metrics [8], evaluated telemedicine broadly without considering program differences [9], or focused solely on implementation without first characterizing existing platforms [10]. Additionally, none of these proposed frameworks were developed in consultation with a panel of stakeholders, nor do they offer a usable instrument for obtaining the information needed for evaluation. The current absence of such a usable instrument – one that rigorously captures the process of telemedicine roll-out and systematically describes existing telemedicine program features – prohibits widespread, practical evaluation of telemedicine programs.

### Objective

Therefore, in the context of a larger United States telemedicine evaluation project, this study aimed to develop a semi-structured survey informed by existing evidence, relevant frameworks, and input from a variety of stakeholders including patients, clinicians and researchers.

## Methods

This study is part of larger study funded by the Patient-Centered Outcomes Research Institute (PCORI) with the objective of describing and evaluating the impact of telemedicine programs in primary care implemented in the wake of the COVID - 19 pandemic. Here, we describe the development of a semi-structured survey to be applied in interviews with primary care practice leaders across the United States. The goal of the survey is to systematically describe practices’ telemedicine features and the process of telemedicine implementation to understand the telemedicine programs’ life cycle.

**Fig. 1:**
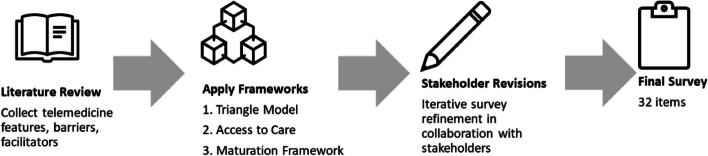
Schematic model of the survey design process

The survey design process included three iterative steps: 1) a literature review of evidence on barriers to and facilitators of telemedicine, 2) application of three different frameworks, and 3) input from various stakeholders on relevance of items and formulation of questions (Fig. 1). Each step is described in more detail below.

### Literature review

Two electronic databases (PubMed and Web of Science) were queried by two researchers for relevant articles published until May 2020. The search strategy included the following keywords: Telemedicine, Telehealth, Primary Care, General Practice, Family Doctor, Primary Health Care, Primary Healthcare, Barrier(s), Facilitator(s), Evaluation, Features, Characteristics. Studies were included if they described (evidence on) features of telemedicine programs in terms of being facilitators of or barriers to telemedicine implementation. Collected features were non-exclusive to a particular participant in the telemedicine process (i.e., factors relating to patients, providers, practice, or technology were all considered).

### Frameworks

The features of telemedicine identified in the previous stage were matched to elements of two theoretical frameworks: the Triangle Model for health information technology evaluation [11] and Penchansky and Thomas’ access to care framework [12]. A third health IT life cycle framework was applied to describe the evolution of telemedicine programs over time [13].

#### Triangle model

**Fig. 2:**
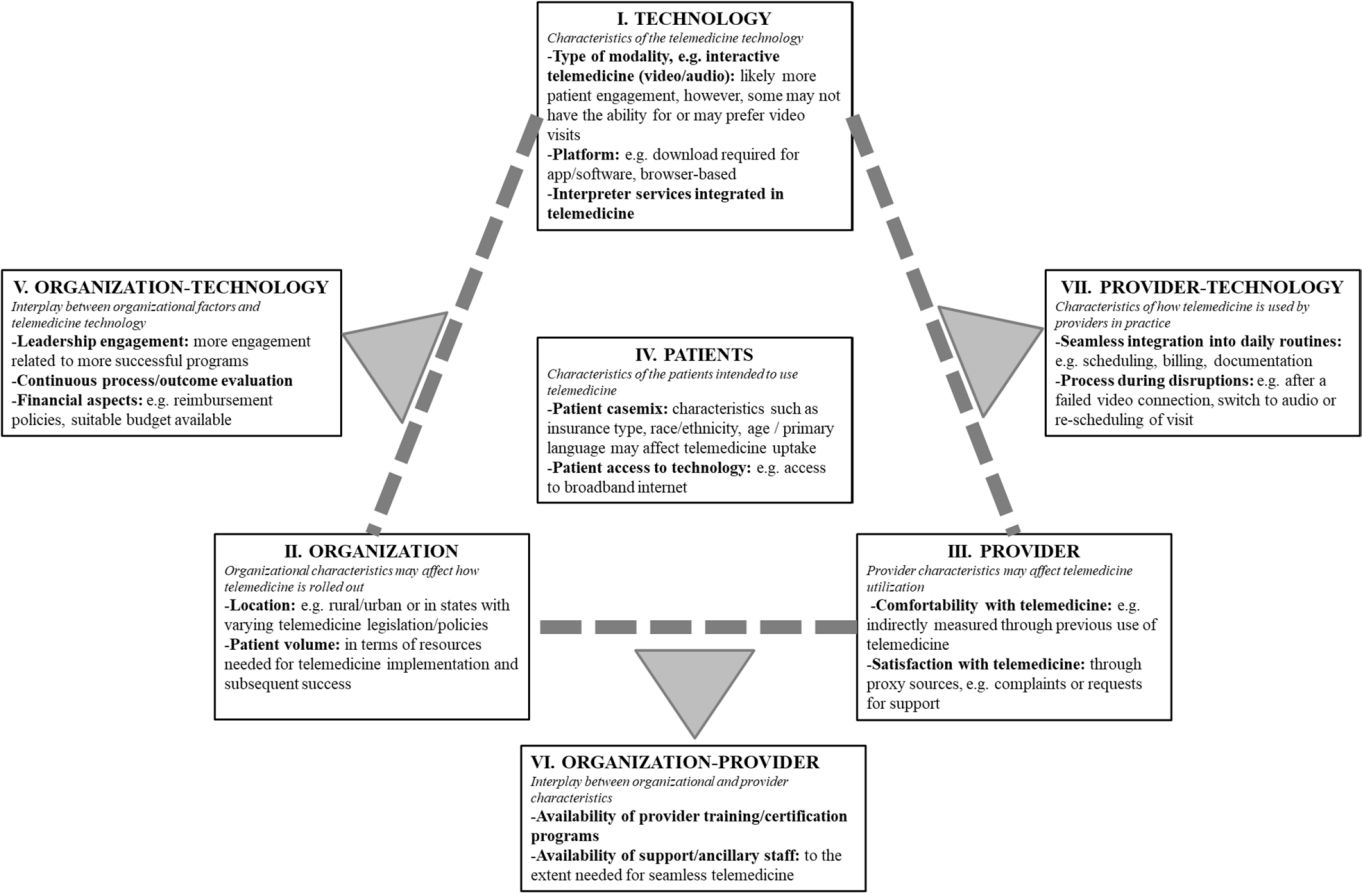
Triangle model modified for telemedicine

The Triangle Model, first developed to evaluate health information technology systems such as the electronic health record (EHR), provides a cross-sectional framework that encourages qualitative and quantitative assessment of structure and process factors of the health information technology [11]. This framework identifies seven domains for evaluation: (I) the technology of interest, (II) the organization that adopts this technology, (III) users of the technology, (IV) the patient population served, (V) the relationships between the organization and the technology, (VI) the relationships between the organization and the providers, and (VII) the relationships between users and the technology. Figure 2 provides an overview and application of this model to the context of telemedicine with telemedicine-specific examples. Application of the Triangle model involved grouping identified features of telemedicine into each domain.

#### Penchansky and Thomas’ access to care framework

This framework conceptualizes access to healthcare in terms of the fit between features of providers and health services, and patient characteristics and expectations [12, 14]. In other words, access is conceived as the interface between potential users/patients and healthcare resources, influenced by features of those who supply and those who utilize the services. We applied it given telemedicine’s theoretical potential to improve access to care by eliminating physical barriers such as distance or allowing more flexibility in terms of scheduling [15, 16]. Access to care elements in this framework include: A) affordability, B) accommodation, C) availability, D) accessibility, and E) acceptability. Application of the access to care framework involved assessing each identified telemedicine feature in terms of their potential to impact access to care based on relevance to each of the aforementioned five access to care elements.

#### Health IT life cycle framework (‘maturation’)

This framework was applied as telemedicine programs were rolled out swiftly given the acute onset of the pandemic, and telemedicine programs gradually "matured" after widespread implementation. The unique and acute nature of the pandemic also almost certainly precluded the types of ongoing formative evaluations thought to be crucial for any health information intervention to achieve its full potential [5, 6]. We aimed to quantify the process of telemedicine program "maturation" using the health IT life cycle framework. While various of such frameworks exist, life cycle phases generally include the following: 1) planning, 2) design and development, 3) implementation and use, and 4) a fully operational phase with monitoring, evaluation, and optimization of the health IT application of interest [3, 4, 13]. Application of the health IT life cycle framework involved querying a perceived point of maturation of a telemedicine program and what telemedicine features in particular were associated with this point of maturation.

### Stakeholder engagement

To support the parent study of telemedicine programs in primary care, a stakeholder board was established using a previously applied methodology [17]. The main goal of this stakeholder board was to provide critical input at various stages of our research. Overall, 15 members (10 female, 5 male) agreed to serve on the board: five patients with chronic diseases (three aged > 65 years), four primary care providers with telemedicine experience, three health informatics experts, one primary care healthcare administrator, two payer administrators, and one patient advocate. Of note, the stakeholder board did not include family members of patients; inclusion of this group would have likely provided further insights as to the experiences of older patients who require assistance with telemedicine. For this survey development project, stakeholders were invited to provide input on 1) items that are important to consider in evaluations of telemedicine programs, and 2) how to formulate these items as survey questions. More specifically, the survey refinement process involved identifying features of telemedicine that were most relevant to patient experiences and practice operations, combining related features into a single survey item, and removing questions that could be answered via quantitative data provided by the participating institutions. A 2-stage feedback process consisted of one formal meeting with the stakeholder board and the research team as well as empowering members to provide asynchronous feedback (through email) in response to each of the four revised versions of the survey. This extensive engagement process with the stakeholder board occurred longitudinally throughout the survey development, and all feedback and associated survey edits were noted and incorporated. This process continued until a final draft of the survey was ready for administration to primary care practice leaders, designed to be completed in 20–25 - min interviews.

The final survey questions were input into REDcap, a secure online application for capturing data and managing databases [18]. Most questions could be answered with ordinal or nominal responses, and respondents were also encouraged to supplement their interviews with narratives, anecdotes, and elaboration.

### Survey administration and data reduction analysis

The developed survey was administered in 20–25-min virtual interviews with primary care practice leaders from four large health systems (Weill Cornell Medicine, Mount Sinai, University of North Carolina, and University of Florida) participating in the Patient-Centered Outcomes Research Institute PCORnet network. Practice leader participation was optional and voluntary.

Data reduction techniques were applied to the collected survey data to determine if there were associations between items of the survey i.e., if items on the survey could be reduced to summary variables / questions. Where needed, continuous variables were transformed into categorical variables; this was either distribution - based or based on natural cutoffs. Levels of categorical variables were inspected manually and re - coded as appropriate. The conversion to categorical variables was a decision that facilitated the data reduction analysis as a means of “pre-summarizing and reducing the data;” the conversion to categorical variables does not preclude the use of continuous data in future uses of this instrument. Finally, the missing values of the continuous variables were imputed using their medians. The dimension reduction technique applied was factor analysis of mixed data (FAMD); here, the representation of continuous variables is constructed as in a principal component analysis (PCA), and categorical variables are constructed as in a multiple correspondence analysis (MCA). Scree plots were used to determine the least number of factors needed to accurately represent the original data. Analyses were conducted in R (R version 3.6.0).

## Results

Following the literature review, 35 telemedicine-related features were identified, i.e., factors potentially associated as barriers to or facilitators of telemedicine implementation. These were categorized into Triangle Model domains, and features were assessed against Penchansky & Thomas’ access-to-care framework elements (Appendix 1). The process of stakeholder board engagement added 25 more items to this preliminary list adding up to a total of 60 features (Table 1).

**Table 1:** Overview of telemedicine features and telemedicine-related features considered to be included in the survey; features are grouped by Triangle model domains. This list is a combination of results from the literature review and feedback from the stakeholder board (see Appendix 1 for an overview of features resulting just from the literature review grouped by Triangle model domains)

**Organization / Practice**	**Providers**	**Technology (Telemedicine)**	**Patients**
Practice Location	Staff Size	Type of telemedicine	Patient Casemix
Urban/Rural	Trainees	Mode of telemedicine access	Patient Access to Technology
Practice Age	Pre-Pandemic Telemedicine	Electronic Health Record Integration	
Practice type (e.g. community, academic)	Pre-Pandemic Satisfaction with Telemedicine	Telemedicine Vendor	
Practice Ownership		Interpreter Services	
Patient Volume			
Patient Casemix			
Pandemic Funding			
Pre-pandemic Telemedicine Use			
**Organization—Technology**		**Provider—Technology**	**Organization—Provider**
Roll - out Processes	Additional Time Needed for Virtual Visit Policy	Scheduling	Provider Training
Acute or Paced Roll-Out	Identity Confirmation	Billing	Support Staff
Extent of Telemedicine Roll-Out	Support Staff Involvement	Documentation	
De Novo or Pre - Existing Telemedicine	Patient Information Follow - up	Check - In Process	
Open/Close Policy of Practices During Pandemic	Data Collection / Continuous Evaluation	Check - Out Process	
Leadership Engagement at Roll - Out	Patient Education / Support	Urgent Visit Policy	
Need -Driven Implementation	Privacy Concerns / Data Security	Ordering of Labs and Imaging Policy	
Telemedicine ‘Champions’	Malpractice Concerns	Productivity	
Telemedicine to Supplement or Substitute In - Person Visits	Licensure Restrictions		
Telemedicine Awareness / Marketing / Outreach	Equipment Cost		
Telemedicine Limited to Subgroups	Reimbursement		
Telemedicine Scheduling Processes	Barriers		
Telemedicine Offered Outside of Traditional Hours	Long - Term Telemedicine Plan		
Disruption of Virtual Visit Policy	Threshold for ER / Urgent Care		
Clarity on Who Qualifies for a Telemedicine Visit	User-Promoter Collaboration		

In the final two rounds of stakeholder board engagement, the research team specifically queried which items would be the most important to include in the survey, while also taking into account a duration of 20–25 min for an interview. The stakeholder board also advised on how questions should be formulated. This resulted in a final survey of 32 items (Table 2; the actual survey can be found in Appendix 2).

**Table 2:** Survey items and questions, response types, and response options (the final REDcap survey can be found in Appendix 2)

#	Feature of interest	Survey Question	Response Type	Response Options (if closed)
1	Respondent Role	Which of the following best describes you?	Closed, mutually exclusive	Administrator; Healthcare provider; Both; Other
2	Practice Name	Which practice or practices can you answer questions about?	Open	
3	Practice Designations	Which of the following applies to your practice?	Closed, non-mutually exclusive	Hospital-based practice; Community practice; Private practice; Solo practice; Federally Qualified Health Center; Affiliated with an academic medical center; Other
4	Staff Size	How many physicians and non-physician staff work at your primary care practice?	Numerical	
5	Medical Residents	Does your practice host medical residents?	Closed, binary	
6	Patient Language	What (estimated) percentage of your patient population does NOT have English as their primary language?	Percentage	
7	Pre - pandemic Telemedicine	Did your practice offer telemedicine services before the start of the COVID-19 pandemic (before March 2020)?	Closed, binary	Yes; No
8	Month of Maturation	Looking back at the roll-out of your practice's telemedicine program, can you pinpoint the month in which you felt your program had matured?	Month—Year	
9	Telemedicine Modality	What type of telemedicine did your practice offer?	Closed, non-mutually exclusive	Interactive / synchronous telemedicine (video and audio); Interactive / synchronous telemedicine (audio - only); Remote patient monitoring
10	Telemedicine Vendor	Which vendor(s) provided telemedicine services?	Open	
11	EHR Integration	What was the relationship between the telemedicine software and the electronic health record (EHR)?	Closed, mutually exclusive	Stand - alone telemedicine platform, NOT INTEGRATED into the EHR; Stand-alone telemedicine program; INTEGRATED into the EHR; Telemedicine platform is the EHR
12	Platform Access	How did patients access telemedicine services?	Closed, non-mutually exclusive	Access through a patient portal; Access without a patient portal
13	Portal Language	If a patient portal was used for telemedicine, was it offered in languages other than English?	Closed, non-mutually exclusive	No; Yes, Spanish; Yes, Mandarin; Yes, Cantonese; Yes, Tagalog (Filipino); Yes, Vietnamese; Yes, Arabic; Yes, other language(s)
14	Interpreter Integration	Were interpreter services available during telemedicine visits?	Closed, binary	Yes; No
15	Patient Education	Which patient education or services did you provide to help patients with telemedicine visits?	Closed, non-mutually exclusive	None; Online material: text; Online material: video; Paper material; Available tech support staff; Phone call ahead of telemedicine visit; Real - time chatbots; Other
16	Visit Limitations	Was telemedicine limited to certain patients, health insurance plans, or visit types?	Closed, non-mutually exclusive	Telemedicine was NOT limited to certain patients, health insurance plans, or visit types; Telemedicine was limited to only patients with certain CHRONIC DISEASES; Telemedicine was limited to only patients with certain HEALTH INSURANCE PLANS; Telemedicine was limited to certain VISIT TYPES only
17	Offered Hours	Were telemedicine visits offered outside of traditional office hours?	Closed, binary	Yes; No
18	Patient Access	How would you rate your patient population's access to technology for telemedicine services?	Closed, 5- level scale	Very poor; Poor; Acceptable; Good; Very good
19	Patient Disparities	How has your practice's telemedicine program impacted disparities in access to care?	Closed, mutually exclusive	It has INCREASED disparities; It has NEITHER decreased NOR increased disparities; It has DECREASED disparities; Don't know
20	Supplement vs. Substitute	Was telemedicine mainly used to supplement or substitute in - person care?	Closed, non-mutually exclusive	Telemedicine was mainly used to SUPPLEMENT in-person care; Telemedicine was mainly used to SUBSTITUTE in-person care; Telemedicine was mainly used to both SUPPLEMENT AND SUBSTITUTE in-person care
21	Scheduling	How easy was it to schedule telemedicine visits?	Closed, 5-level scale	1—most difficult; 2; 3; 4; 5—easiest
22	Billing	How easy was it to bill for telemedicine visits?	Closed, 5-level scale	1—most difficult; 2; 3; 4; 5—easiest
23	Visit Documentation	How easy was it to document the content of telemedicine visits?	Closed, 5-level scale	1—most difficult; 2; 3; 4; 5—easiest
24	Reimbursement	How easy was it to get reimbursed for telemedicine visits?	Closed, 5-level scale	1—most difficult; 2; 3; 4; 5—easiest
25	Check - in	Did the CHECK-IN process for a telemedicine visit involve an interaction with a remote support staff member?	Closed, mutually exclusive	Never; Sometimes; Always
26	Check - out	Did the CHECK-OUT process for a telemedicine visit involve an interaction with a remote support staff member?	Closed, mutually exclusive	Never; Sometimes; Always
27	Technology Disruptions	According to your estimation, how often were telemedicine visits disrupted due to a technological issue?	Closed, 5-level scale	Never; Seldom; Sometimes; Frequently; Always
28	Provider Training	Did your practice offer training or certification before authorizing providers to use telemedicine?	Closed, non-mutually exclusive	No, there was no formalized provider certification program at our practice; Yes, an online training; Yes, an in-person training; Yes, paper or electronic handouts; Yes, other
29	Support Staff	Please rate the following statement: there was enough support staff available for our practice's telemedicine program	Closed, 5-level scale	Strongly disagree; Disagree; Undecided; Agree; Strongly Agree
30	Financial Investment	Looking back, how much did your practice invest financially in these aspects of your telemedicine program? (staff equipment, patient equipment, training, additional staff, tech support, vendor)	Closed, mutually exclusive	No investment; Minimal investment; Moderate investment; Significant investmentl; Don’t know / unsure
31	Open - Ended	Tell us more about the process of telemedicine roll-out in your practice, any specific additional things that you feel might be important for us to understand?	Open	
32	Future Telemedicine	Which of the following best describes your future plans for telemedicine in your practice after the current public health emergency ends?	Closed, mutually exclusive	We plan to SCALE DOWN our telemedicine program and return to in-person care as much as possible; We plan to continue to offer telemedicine at the SAME AMOUNT as during the pandemic; We plan to EXPAND our telemedicine program; Unsure at this time

The final domains covered practice characteristics, patient population characteristics, technical features, barriers to care, patient services, operational processes, financial investment, and future plans for telemedicine. Each item in the survey also included opportunities to record associated qualitative information. In order to systematically describe the evolution of telemedicine programs (and taking into account the health IT life cycle framework) a question was included on the perceived point of maturation of a telemedicine program. Questions 9–29 on the features of the telemedicine program were asked twice, once for the program at the onset of the pandemic and a second time at the point identified as telemedicine program maturation.

The survey was administered to 33 practice leaders representing 100 unique primary care practices across four states. Characteristics of the result can be found in Table 3. From this data, 18 continuous and 40 categorical variables were extracted for data reduction analysis. Scree plots (Appendices 3 and 4) created from the data reduction analyses indicated that there was no clear “elbow” with a rather smooth plot, i.e., no indication for a suitable number of factors to retain. This was the first indication against the use of a data dimension reduction approach. To further explore, the top ten variables contributing to each of the first four dimensions/factors, representing a total of 33 variables (instead of the 58 variables used in the previous analysis), were selected and the same analysis was performed on the smaller set. Resulting scree plots showed a similar pattern, i.e., no clear elbow.

**Table 3:** Telemedicine features stratified by March 2020 and point of telemedicine program maturation (*n* = 100 practices)

Item	March 2020 Practice Counts
**Telemedicine technology**	
***Type of telemedicine offered***	
- Video and audio	99
- Audio only	64
- Remote patient monitoring	27
***Telemedicine - EHR integration***	
- No integration	8
- Different systems, integrated	61
- Same system	31
**Access to telemedicine**	
***Portal used for telemedicine access***	
- Yes	87
***Portal offered in language other than English***	
- Yes	8
***Interpreter services integrated***	
- Yes	27
***Available patient education / services***	
- Online text	78
- Online video	42
- Paper material	59
- Tech support	78
- Call ahead of visit	43
- Other (incl. real - time staff / medical students calling ahead, doing vitals, training at intake / onsite)	14
***Telemedicine restricted to:***	
- No restrictions	53
- Patients with certain diseases	0
- Patients with certain insurance types	22
- Certain visit types	32
***Telemedicine offered outside traditional hours***	
- Yes	36
***Patients’ access to telemedicine tech***	
- Very poor	2
- Poor	16
- Acceptable	19
- Good	39
- Very good	24
***Telemedicine's impact on disparities***	
- Increased	12
- Neither	50
- Decreased	35
**Telemedicine in daily practice**	
***Telemedicine as supplement / substitute for in-person care***	
- Supplement	30
- Substitute	56
- Both	14
***Ease of scheduling telemedicine visits***	
Mean (1–5, 1 = difficult, 5 = easiest)	2.98
***Ease of billing for telemedicine***	
Mean (1–5, 1 = difficult, 5 = easiest)	3.09
***Ease of documentation during telemedicine visits***	
Mean (1–5, 1 = difficult, 5 = easiest)	3.75
***Ease of reimbursement for telemedicine***	
Mean (1–5, 1 = difficult, 5 = easiest)	2.84
***- Interaction with staff during check-in***	
- Never	23
- Sometimes	28
- Always	49
***- Interaction with staff during check-out***	
- Never	57
- Sometimes	37
- Always	6
***Telemedicine visit disrupted due to tech issue***	
- Never	0
- Seldom	23
- Sometimes	53
- Frequently	22
- Always	2
**Certification and support staff**	
***Provider training / certif. offered***	
- None	13
- Online training	48
- In-person training	63
- Paper / electronic handouts	39
***Enough support staff for telemedicine***	
- Strongly disagree	3
- Disagree	31
- Undecided	29
- Agree	33
- Strongly agree	4

## Discussion

This article described the process of developing a semi - structured survey to be used in a larger study characterizing telemedicine programs in primary care rolled out in response to the COVID-19 pandemic. Via an iterative process that involved querying existing evidence, the application of relevant frameworks, and extensive stakeholder input, a 32-item tool was developed to systematically quantify telemedicine programs vis-à-vis telemedicine and telemedicine-related features as well as the process of telemedicine maturation, while also allowing room for non-structured responses to fully capture practice-specific telemedicine experiences. This tool will facilitate not only evaluations but also comparisons between telemedicine programs across the United States, particularly those that were deployed at the pandemic onset.

The roll-out of telemedicine across the pandemic was an unprecedented implementation of a technology in healthcare. For this reason, there is no gold standard or accepted truth for the question of this study: how telemedicine programs were rolled-out in primary care practices. However, the data reduction techniques applied provides reassurance that the items included in the survey were not redundant and could not be summarized with fewer variables. Each item therefore contributes value to the overall tool; the tool cannot be shortened without losing unique information on practices.

The strength of the developed survey tool lies in the inclusion of a thorough overview of existing evidence, the application of relevant frameworks and, importantly, inclusion of stakeholder feedback, which are crucial recommended steps in survey development [19, 20]. These steps, particularly the latter, are designed to ensure items’ relevance, content validity and clarity. The current lack of an instrument to systematically measure telemedicine programs justifies follow-up research on improving and modifying our survey. For example, future work could include validation of the survey and modifying the survey (e.g., shortening through data reduction techniques or modifying it based on the intended respondent). Following development, to date, this tool has been used in 33 interviews with primary care practice leaders across the United States, and important lessons related to wording of questions and time needed for interviews have been learned. Results of this ongoing process will be reported with the completion of each step.

Lacking systematic and standardized evaluations of health IT interventions is a known limitation in the health IT evaluation literature as it has been identified as a major barrier to between - study comparisons, generalizability of evaluation results, and importantly, reproducibility [21, 22]. This problem is compounded by various factors including heterogeneity in terms of the respective health IT intervention’s features, implementation context, organizational context, site - specific user characteristics, and more generally a lack of standard for best practices on reporting results of health IT intervention evaluations, even though numerous frameworks exist [23, 24]. Most of these limitations are relevant to the current telemedicine literature in primary care especially given the fast pace of roll-out of such programs with the onset of the COVID - 19 pandemic [25–29]. Indeed, most primary care telemedicine evaluation studies - specifically those published since the pandemic onset - either describe single institution (or single health system) experiences or do not clearly report the full extent of telemedicine features employed, with data missing on important contextual factors such as provider training in telemedicine, patient populations’ access to telemedicine technology, or support for patients opting to have a telemedicine visit [25–29]. Moreover, data is lacking on the process of maturation of telemedicine programs as this may have been different between practices in early hotspots of the pandemic (e.g. New York City region) compared to practices in other geographic areas. Perhaps the best parallel can be drawn with the fast adoption of EHR systems, accelerated in part by federally mandated programs such as Meaningful Use (currently known as Promoting Interoperability) supported through the Health Information Technology for Economic and Clinical Health (HITECH) Act [30]. Here, similar concerns have been noted such as lack of contextual information, non-standardized reporting and uncertainty regarding generalization [31, 32]. Application of the survey tool developed in the current study aims to address the aforementioned limitations of the current telemedicine literature, specifically in case of conflicting findings. One such example concerns the hypothesized impact of telemedicine on disparities in access to care; here, some studies point to telemedicine’s potential to reduce disparities [33] while others have noted concerns on a potential increase in disparities [1]. Employing a tool to describe telemedicine programs and their context in a systematic way may prove useful in explaining the mechanism behind such contradicting results.

One important feature of the developed survey is the quantification of the maturation process of telemedicine programs, a crucial aspect to take into account when comparing telemedicine program evaluations, as recently noted [34]. Indeed, various health systems had adopted telemedicine in some form before the pandemic, and it is likely that they were better equipped to manage and underwent different maturation processes relative to health systems that suddenly needed to implement telemedicine programs and infrastructure from scratch at the pandemic onset. More research is needed to quantify the head start these early adopters enjoyed and how this impacted patients’ access to care. Moreover, the dynamic nature of the pandemic – including subsequent COVID - 19 variants and surges coming and going – may require a telemedicine-specific maturation framework as practices and health systems may scale up telemedicine programs and features in response to surges in COVID - 19 cases, with the reverse happening during steady-states.

In part based on stakeholder feedback, our survey explicitly allows for qualitative responses outside of the structured questions. This was deliberate as we expected there to be unique practice-specific and health system-specific experiences regarding the roll-out of telemedicine programs, particularly as a result of the acute need to rapidly deploy and scale individual telemedicine programs. Moreover, other factors such as a practice’s or health system’s culture are hard to capture with structured questions or quantitative data. As stated, we are currently in the process of applying the survey to primary care practice leaders across the United States and extracted qualitative data may also inform future survey modifications.

Importantly, the developed survey does not include any items or variables to assess the quality or value of telemedicine programs. This is an artifact of the timing of this study, as initial steps toward developing the survey began in the immediate post-pandemic era. While value and quality metrics have important implications, many telemedicine programs were far too young to retrieve any meaningful outcomes data. There have been numerous publications demonstrating mixed results regarding specific telemedicine outcomes since the pandemic [35–37], and future work examining telemedicine outcomes is certainly needed. Data gleaned from the use of our proposed instrument can provide a comprehensive picture of specific telemedicine programs, necessary context for any future quality or value assessment.

This study has limitations. The survey has not yet been validated. Performance of the survey tool, as it relates to obtaining adequate telemedicine details, may differ in other practices and health systems. A different or larger group of stakeholders might have provided different input. Here, we aimed to balance the practicality of efficiently executing the research against theoretical perfection. Additionally, our tool was developed to be used in 20–25-min virtual interviews with primary care practice leaders; depending on the study objective of interest, intended type of respondent and time available for interviews, this exact tool may not be as useful in other telemedicine research and may require project - specific modifications. Finally, the telemedicine landscape has continued to evolve and change over the course of this project. While this instrument was designed in a way to make it applicable to non - pandemic era telemedicine programs in addition to pandemic - implemented programs, the notable changes in telemedicine utilization [38], guidelines [39], regulation [40], and reimbursement [41] should be noted.

The tool developed in this study has significant implications at the practice level. This standardized evaluation of telemedicine programs provides a framework for practice leaders to assess, compare, and iterate telemedicine programs. Using this tool, practices will be able to undergo continuous quality improvement initiatives more effectively as this tool offers a holistic assessment of programs, as supported by the rigorous development process documented in this manuscript. For practices that are seeking to roll - out new or re - designed telemedicine programs, the tool can act as a comprehensive list of characteristics of telemedicine programs. Administrators of telemedicine programs could reference this survey to ensure that they have considered all of the included features of their telemedicine programs. Additionally, this tool has the ability to highlight factors associated with telemedicine program evolution. Understanding and targeting these areas may accelerate the refinement of telemedicine programs moving forward.

In conclusion, in this paper we describe the process of developing a 32-item semi-structured survey to standardize the description of primary care telemedicine programs in terms of features as well as maturation process. This tool will facilitate not only evaluations but also comparisons between telemedicine programs across the United States, particularly those that were deployed at the pandemic onset.

### Supplementary Information

**Additional file 1: Appendix 1.** Initially identified features of telemedicine (and healthcare delivery context) based on a review of existing evidence, categorized by Triangle model domains with each feature assessed against Penchansky & Thomas ‘access - to - care’ framework elements.

**Additional file 2: Appendix 2.** Final survey.

**Additional file 3: Appendix 3. **Scree plot; eigenvalues (y-axis) plotted against factors/principal components (x-axis).

**Additional file 4: Appendix 4. **Scree plot; explained variance (y-axis) plotted against factors/principal components (x-axis).

## Data Availability

This data describes the development of a survey which is provided in its entirely in the supplementary files.
