# Supplementary material for: Development of a novel instrument to characterize telemedicine programs in primary care
Source: BMC Health Serv Res. 2023 Nov 17;23:1274. doi: 10.1186/s12913-023-10130-5 (PMC10657014; doi:10.1186/s12913-023-10130-5)
Supplement: Supplementary file 1 — Additional file 1: Appendix 1. Initially identified features of telemedicine (and healthcare delivery context) based on a review of existing evidence, categorized by Triangle model domains with each feature assessed against Penchansky & Thomas ‘access - to - care’ framework elements. [file 12913_2023_10130_MOESM1_ESM.docx]

**APPENDIX I.** **Initially identified features of telemedicine (and healthcare delivery context) based on a review of existing evidence, categorized by Triangle model domains with each feature assessed against Penchansky & Thomas ‘access-to-care’ framework elements.**

Candidate telemedicine domains (**I-VII**) and sub-domains to be included in our survey based on literature review; domains are based on the Triangle Model evaluation framework for health IT (1). References (mostly reviews) are provided for each sub-domain indicating a role in patient outcomes or successful implementation of a telemedicine program. For a subset of sub-domains, relevant Penchansky & Thomas ‘acces-to-care’ framework elements (2, 3) are noted that indicate the potential for care access improvement related to these sub-domains. These include: **A)** affordability, **B)** accommodation, **C)** availability, **D)** accessibility, and **E)** acceptability.

|  | **Evidence indicating positive effect on outcomes / Successful implementation** | **Relevant Penchansky & Thomas ‘access-to-care’ framework elements** |
| --- | --- | --- |
| **I. ORGANIZATION (PRIMARY CARE PRACTICE)** |  |  |
| -Location (rural/urban, in states with varying legislation/policies on telemedicine) | (4,5) | A / D |
| -Patient volume | (6,7) | C |
| -Ownership (e.g. health system-owned/physician-owned) | (5) | B |
| -Practice type (e.g. community, academic, FQHC) | (5) | B |
| -Provider mix (faculty/trainees)  [also under **II. PROVIDER**] | (8) | B / C |
| -Patient mix (insurance, race/ethnicity, age / primary language)  [also under **IV. PATIENTS**] | (9) | A / E |
| **II. PROVIDER** |  |  |
| -Comfortable with telemedicine (e.g. indirectly measured through previous use of telemedicine in practice) | (10) [previous telemedicine use a negative impact] | - |
| -Provider satisfaction with telemedicine (through proxy sources such as complaints or requests for support) | (11) | - |
| -Provider trainee status (faculty/trainee)  [also under **I. ORGANIZATION**] | (8) |  |
| **III. TECHNOLOGY (TELEMEDICINE)** |  |  |
| *Type* |  |  |
| -Remote monitoring | (12-14) (15-17) | B / C / D |
| -Store-and-forward | (16-17) | B / C / D |
| -Interactive telemedicine (video/audio) | (13,16.17) | B / C / D |
| -Interactive telemedicine (audio-only) | (14-17) | B / C / D |
| *Mode* |  |  |
| -Patient portal | (18) [portal potential negative impact] (19) | B |
| -App/Software | (20) | B |
|  |  |  |
| -Electronic medical record integration | (4,21) | - |
| -Interpreter services (general availability and mode of operation during telemedicine visits) | (22) | B / C / E |
| **IV. PATIENTS** |  |  |
| -Patient mix (insurance, race/ethnicity, age / primary language)  [also under **I. ORGANIZATION**] | (9) | A / E |
| -Patient access to technology | (4,23) | B / E |
| **V. ORGANIZATION--TECHNOLOGY** |  |  |
| -Leadership engagement | (24) | - |
| -Needs driven implementation of telemedicine | (4,9,23,25) | B / C |
| -Supplementation or substitution of usual care (e.g. in-person visit) | (16) | - |
| -Identification of ‘champions’ | (4,23,25,26) | - |
| -Raising awareness among (potential) patients / marketing / outreach (e.g. to get registered onto the electronic medical record platform) | (4) | B / E |
| -Clarity on candidate criteria (‘who is a potential candidate for telemedicine?’) | (4,27) | B |
| -Proactive/pre-emptive vs. reactive scheduling of telemedicine visits | (28) | B / C |
| -Continuous evaluation of data (processes and outcomes) | (24,25) | - |
| -Availability outside of office hours | (29) | B |
| -Do novo implementation or expansion of telemedicine | (4) | - |
| -Roll-out characteristics (acute/spread out) | (23,24) | A / B |
| -Long-term plan (e.g. future expansion) |  | C |
| -Disruption of services (e.g. during encounter) | (21,27) (4,24) | B / C |
| -Patient support services / education (e.g. patient preparation through online resources, on the expected process, trouble shooting mechanisms, instructions in case of disruptions during the encounter, etc.) | (4,21,23,25,27) | B / C / E |
| -Data security | (4,21) |  |
| -Financial aspects of telemedicine / reimbursement (what is reimbursed and for who and does it matter in terms of telemedicine practice?) / Suitable budget available (e.g. application for FCC COVID-19 telemedicine funding) | (4,5,23,25) | A / C / E |
| -Collaboration between promoters and users | (9) | B |
| **VI. PROVIDER--TECHNOLOGY** |  |  |
| -Seamless integration into daily routines including services such as scheduling, billing, documentation, check-in/-out process , handling of urgent visits, ordering of laboratory tests/imaging | (4,5,9,23,24) | B |
| **VII. ORGANIZATION--PROVIDER** |  |  |
| -Provider training | (23,26) (4,24) | - |
| -Support staff | (4,5,21,23,26,27) | - |

**References**

**1.** Ancker JS, Kern LM, Abramson E, Kaushal R. The Triangle Model for evaluating the effect of health information technology on healthcare quality and safety. J Am Med Inform Assoc 2012;19:61-5.

**2.** Penchansky R, Thomas JW. The concept of access: definition and relationship to consumer satisfaction. Med Care 1981;19:127-40.

**3.** Ricketts TC, Goldsmith LJ. Access in health services research: the battle of the frameworks. Nurs Outlook 2005;53:274-80.

**4.** Broens TH, Huis in't Veld RM, Vollenbroek-Hutten MM, Hermens HJ, van Halteren AT, Nieuwenhuis LJ. Determinants of successful telemedicine implementations: a literature study. J Telemed Telecare 2007;13:303-9.

**5.** Ranganathan C, Balaji S. Key Factors Affecting the Adoption of Telemedicine by Ambulatory Clinics: Insights from a Statewide Survey. Telemed J E Health 2020;26:218-25.

**6.** Breslow MJ, Rosenfeld BA, Doerfler M, Burke G, Yates G, Stone DJ, Tomaszewicz P, Hochman R, Plocher DW. Effect of a multiple-site intensive care unit telemedicine program on clinical and economic outcomes: an alternative paradigm for intensivist staffing. Crit Care Med 2004;32:31-8.

**7.** Agha Z, Schapira RM, Maker AH. Cost effectiveness of telemedicine for the delivery of outpatient pulmonary care to a rural population. Telemed J E Health 2002;8:281-91.

**8.** Sartori DJ, Olsen S, Weinshel E, Zabar SR. Preparing trainees for telemedicine: a virtual OSCE pilot. Med Educ 2019;53:517-8.

**9.** Obstfelder A, Engeseth KH, Wynn R. Characteristics of successfully implemented telemedical applications. Implement Sci 2007;2:25.

**10.** Hanson D, Calhoun J, Smith D. Changes in provider attitudes toward telemedicine. Telemed J E Health 2009;15:39-43.

**11.** Whitten P, Love B. Patient and provider satisfaction with the use of telemedicine: overview and rationale for cautious enthusiasm. J Postgrad Med 2005;51:294-300.

**12.** Steventon A, Bardsley M, Billings J, Dixon J, Doll H, Hirani S, Cartwright M, Rixon L, Knapp M, Henderson C, Rogers A, Fitzpatrick R, Hendy J, Newman S, Whole System Demonstrator Evaluation T. Effect of telehealth on use of secondary care and mortality: findings from the Whole System Demonstrator cluster randomised trial. BMJ 2012;344:e3874.

**13.** Hopp F, Woodbridge P, Subramanian U, Copeland L, Smith D, Lowery J. Outcomes associated with a home care telehealth intervention. Telemed J E Health 2006;12:297-307.

**14.** Conway A, Inglis SC, Clark RA. Effective technologies for noninvasive remote monitoring in heart failure. Telemed J E Health 2014;20:531-8.

**15.** Inglis SC, Clark RA, Dierckx R, Prieto-Merino D, Cleland JG. Structured telephone support or non-invasive telemonitoring for patients with heart failure. Cochrane Database Syst Rev 2015:CD007228.

**16.** Flodgren G, Rachas A, Farmer AJ, Inzitari M, Shepperd S. Interactive telemedicine: effects on professional practice and health care outcomes. Cochrane Database Syst Rev 2015:CD002098.

**17.** Totten AM, Womack DM, Eden KB, McDonagh MS, Griffin JC, Grusing S, Hersh WR. Telehealth: Mapping the Evidence for Patient Outcomes From Systematic Reviews Rockville (MD), 2016.

**18.** Jansen-Kosterink S, Dekker-van Weering M, van Velsen L. Patient acceptance of a telemedicine service for rehabilitation care: A focus group study. Int J Med Inform 2019;125:22-9.

**19.** Faruque LI, Wiebe N, Ehteshami-Afshar A, Liu Y, Dianati-Maleki N, Hemmelgarn BR, Manns BJ, Tonelli M, Alberta Kidney Disease N. Effect of telemedicine on glycated hemoglobin in diabetes: a systematic review and meta-analysis of randomized trials. CMAJ 2017;189:E341-E64.

**20.** Almathami HKY, Win KT, Vlahu-Gjorgievska E. Barriers and Facilitators That Influence Telemedicine-Based, Real-Time, Online Consultation at Patients' Homes: Systematic Literature Review. J Med Internet Res 2020;22:e16407.

**21.** Sanders C, Rogers A, Bowen R, Bower P, Hirani S, Cartwright M, Fitzpatrick R, Knapp M, Barlow J, Hendy J, Chrysanthaki T, Bardsley M, Newman SP. Exploring barriers to participation and adoption of telehealth and telecare within the Whole System Demonstrator trial: a qualitative study. BMC Health Serv Res 2012;12:220.

**22.** Schulz TR, Richards M, Gasko H, Lohrey J, Hibbert ME, Biggs BA. Telehealth: experience of the first 120 consultations delivered from a new refugee telehealth clinic. Intern Med J 2014;44:981-5.

**23.** Brebner JA, Brebner EM, Ruddick-Bracken H. Experience-based guidelines for the implementation of telemedicine services. J Telemed Telecare 2005;11 Suppl 1:3-5.

**24.** Stevenson L, Ball S, Haverhals LM, Aron DC, Lowery J. Evaluation of a national telemedicine initiative in the Veterans Health Administration: Factors associated with successful implementation. J Telemed Telecare 2018;24:168-78.

**25.** Ellimoottil C, An L, Moyer M, Sossong S, Hollander JE. Challenges And Opportunities Faced By Large Health Systems Implementing Telehealth. Health Aff (Millwood) 2018;37:1955-9.

**26.** Prendergast M, Honey M. The Barriers and Facilitators for Nurse Educators Using Telehealth for Education. Stud Health Technol Inform 2019;264:1323-6.

**27.** Fairbrother P, Ure J, Hanley J, McCloughan L, Denvir M, Sheikh A, McKinstry B, Telescot programme t. Telemonitoring for chronic heart failure: the views of patients and healthcare professionals - a qualitative study. J Clin Nurs 2014;23:132-44.

**28.** Porath A, Irony A, Borobick AS, Nasser S, Malachi A, Fund N, Kaufman G. Maccabi proactive Telecare Center for chronic conditions - the care of frail elderly patients. Isr J Health Policy Res 2017;6:68.

**29.** Due-Christensen M, Kaldan G, Almdal TP, Glindorf M, Nielsen KE, Zoffmann V. Out-of-office hours nurse-driven acute telephone counselling service in a large diabetes outpatient clinic: A mixed methods evaluation. Patient Educ Couns 2015;98:890-4.
