# Supplementary figures and images for: Development of a novel instrument to characterize telemedicine programs in primary care
Source: BMC Health Serv Res. 2023 Nov 17;23:1274. doi: 10.1186/s12913-023-10130-5 (PMC10657014; doi:10.1186/s12913-023-10130-5)

**Appendix II.** **Final survey**


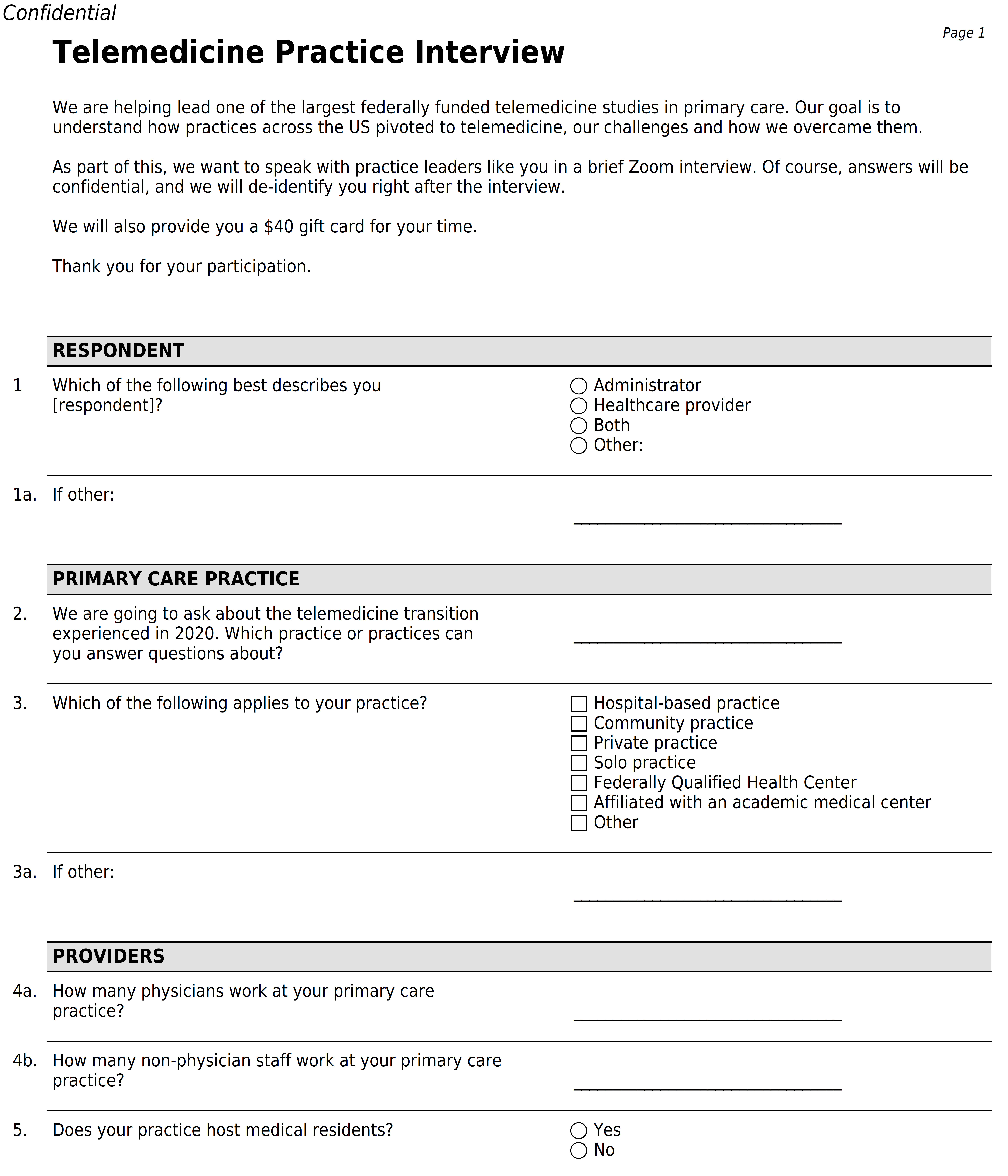


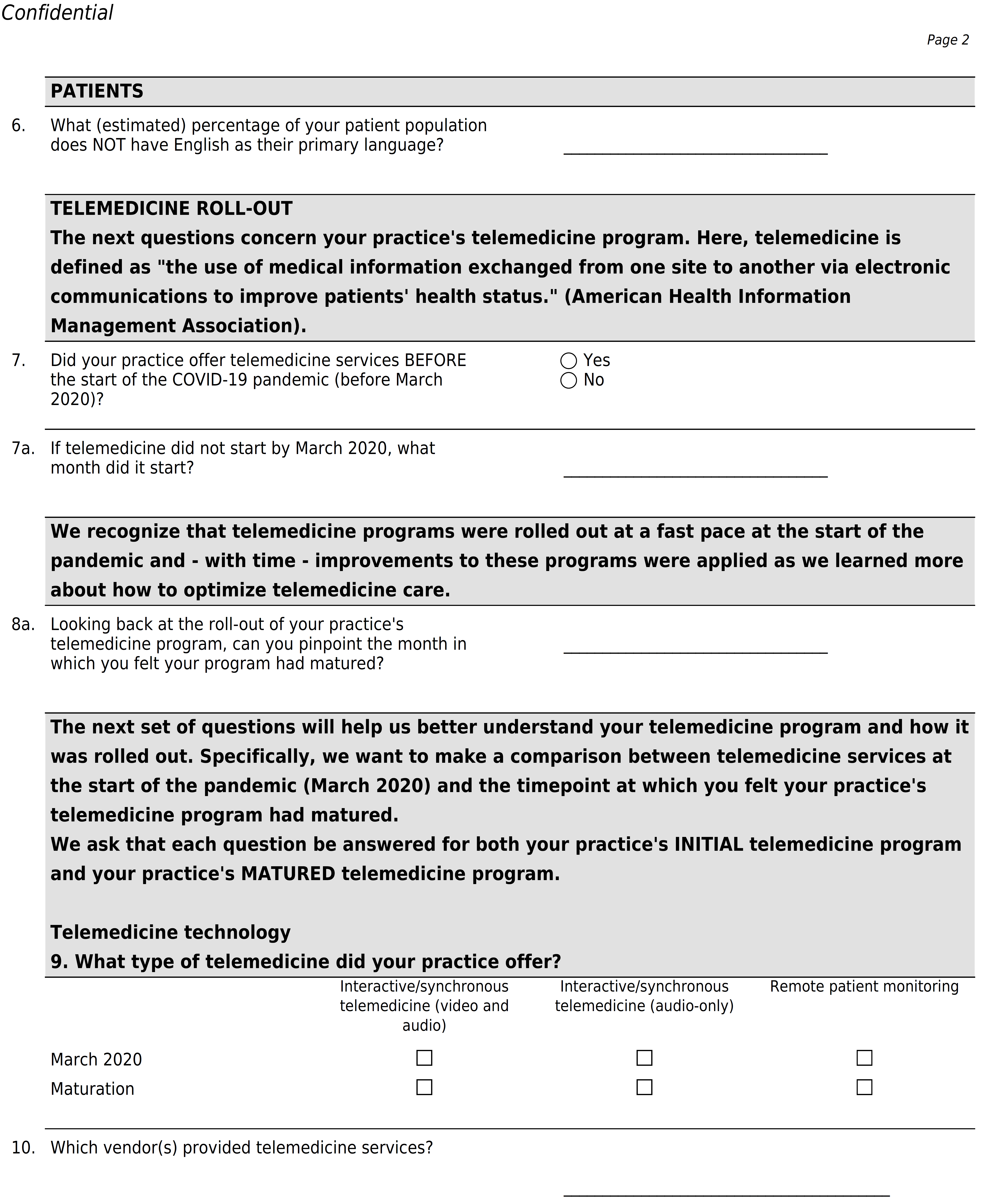


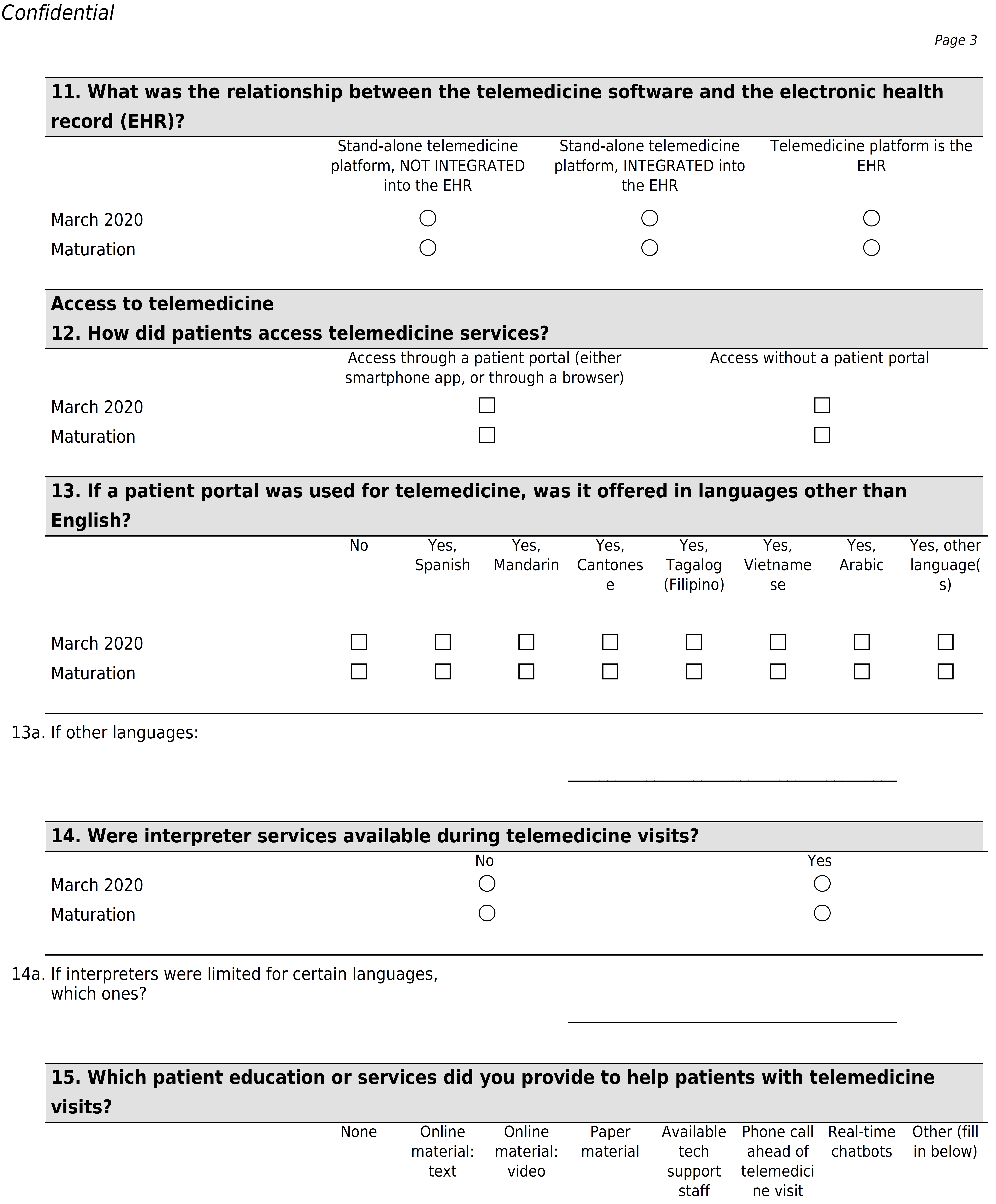


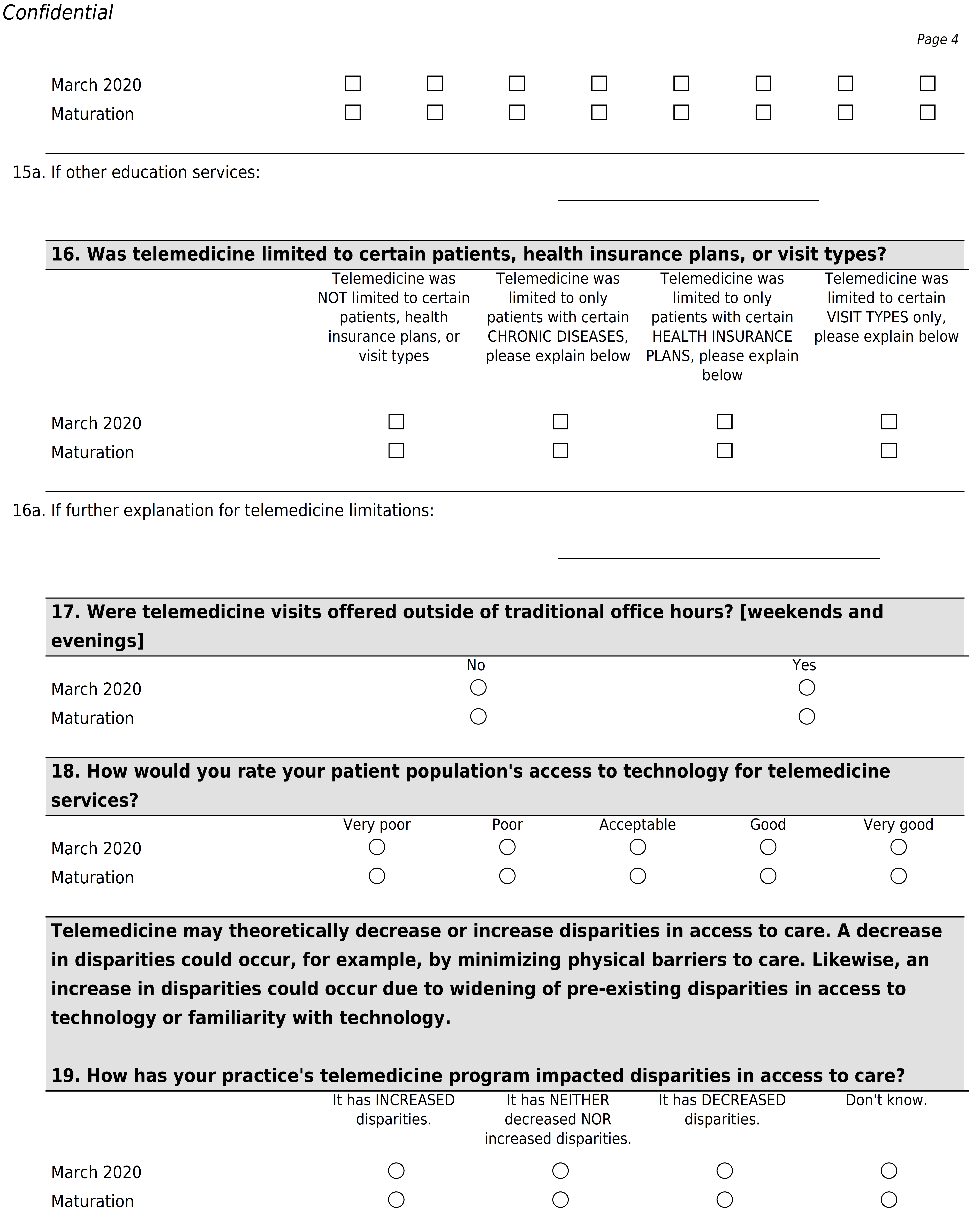


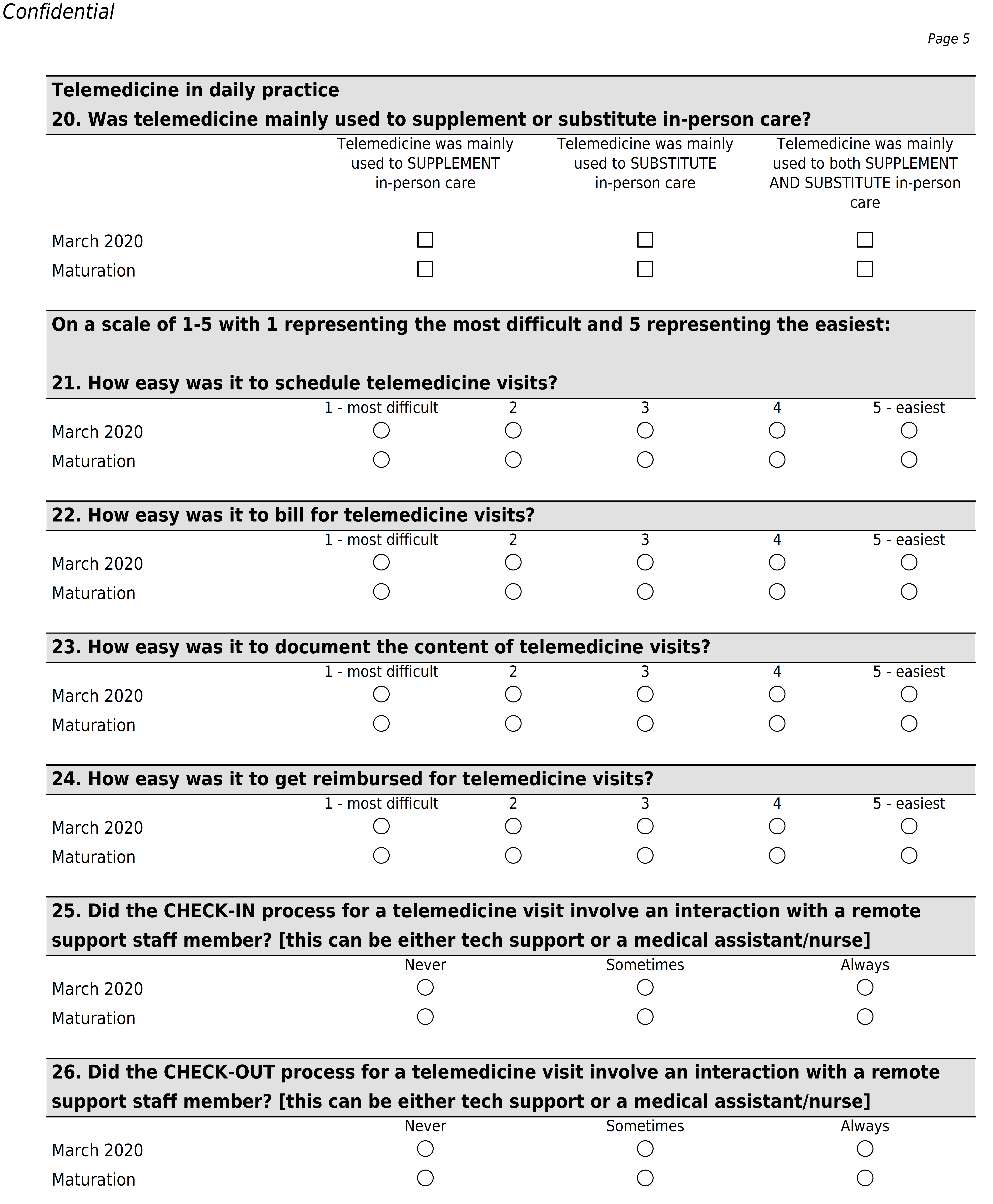


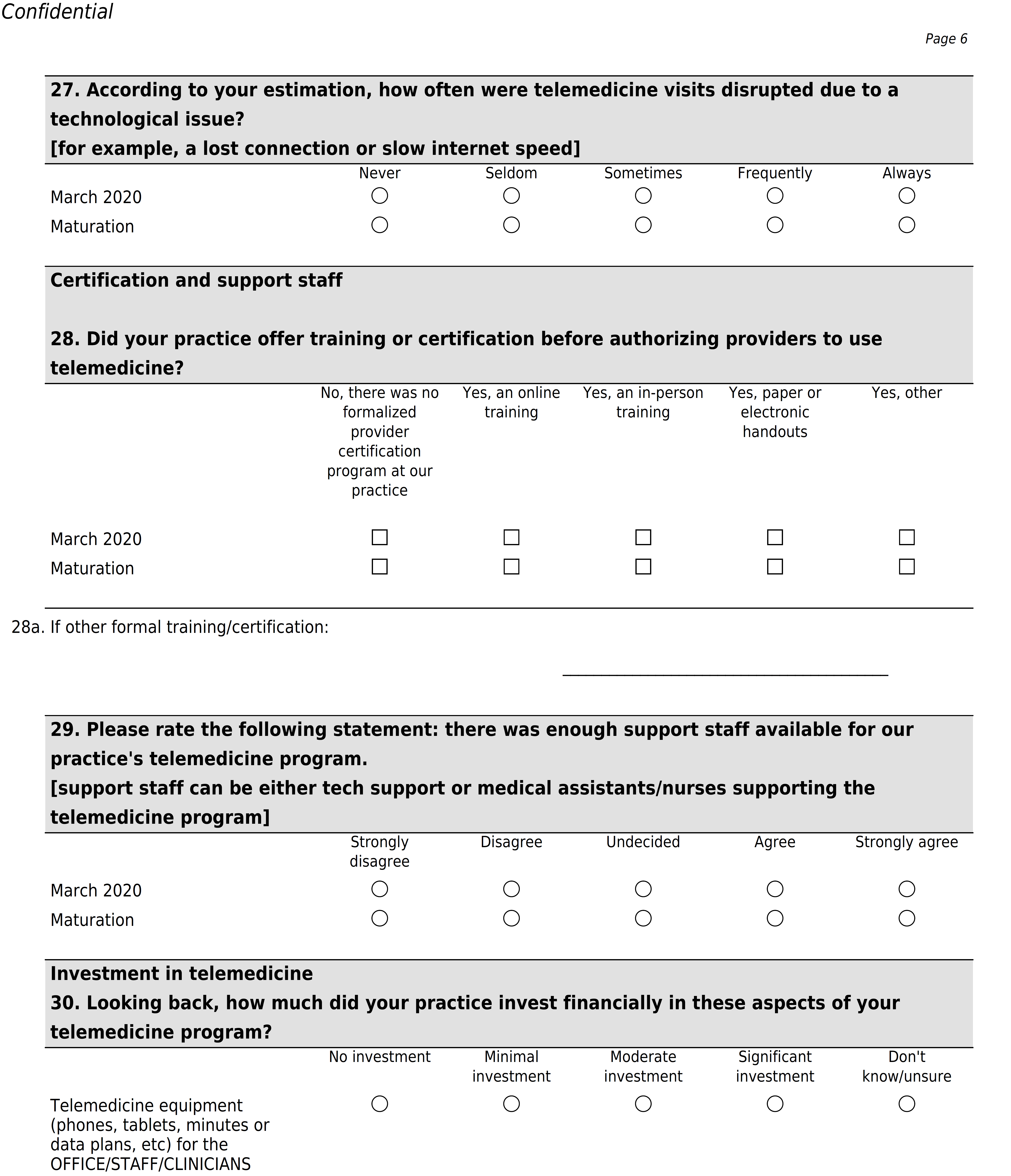


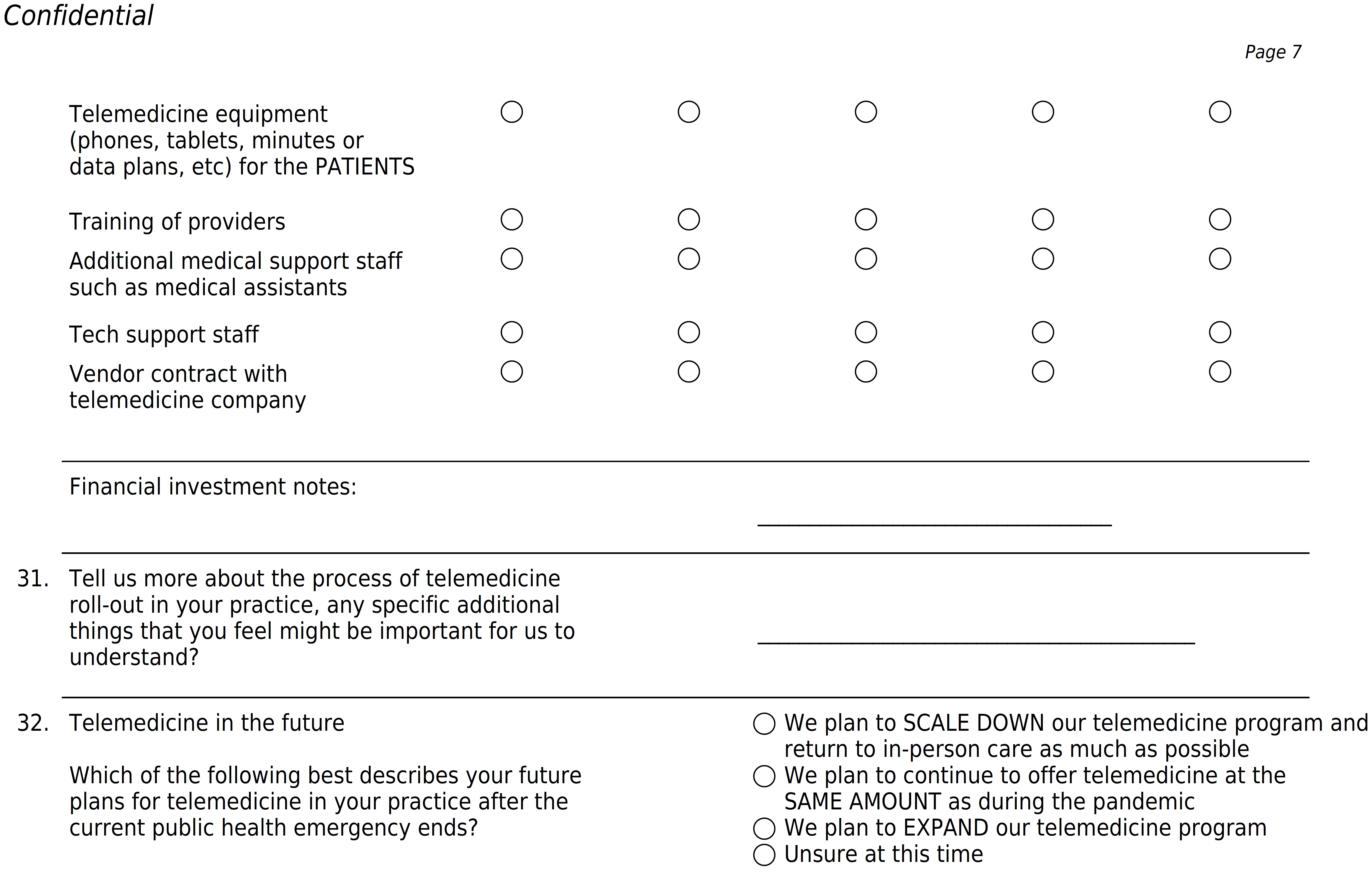

Supplement: Supplementary file 2 — Additional file 2: Appendix 2. Final survey. [file 12913_2023_10130_MOESM2_ESM.docx]
