# Supplementary material for: Development of a novel instrument to characterize telemedicine programs in primary care
Source: BMC Health Serv Res. 2023 Nov 17;23:1274. doi: 10.1186/s12913-023-10130-5 (PMC10657014; doi:10.1186/s12913-023-10130-5)

**Appendix III.** Scree plot; eigenvalues (y-axis) plotted against factors/principal components (x-axis).


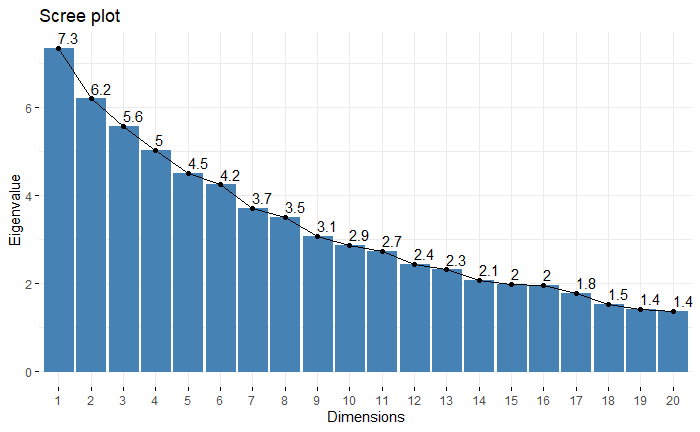

Supplement: Supplementary file 3 — Additional file 3: Appendix 3. Scree plot; eigenvalues (y-axis) plotted against factors/principal components (x-axis). [file 12913_2023_10130_MOESM3_ESM.docx]
