# Supplementary material for: Development of a novel instrument to characterize telemedicine programs in primary care
Source: BMC Health Serv Res. 2023 Nov 17;23:1274. doi: 10.1186/s12913-023-10130-5 (PMC10657014; doi:10.1186/s12913-023-10130-5)

**Appendix IV.** Scree plot; explained variance (y-axis) plotted against factors/principal components (x-axis).


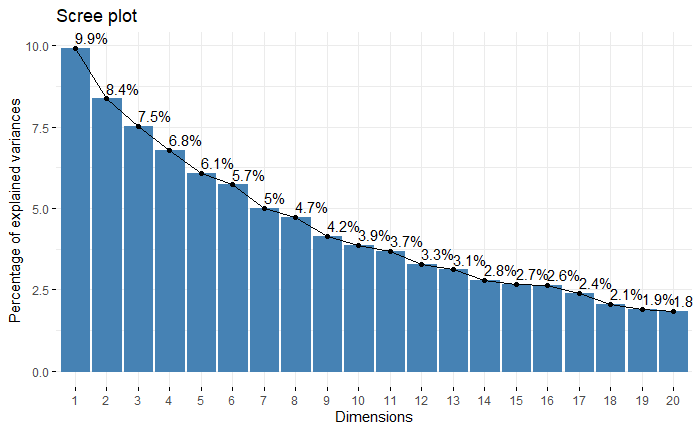

Supplement: Supplementary file 4 — Additional file 4: Appendix 4. Scree plot; explained variance (y-axis) plotted against factors/principal components (x-axis). [file 12913_2023_10130_MOESM4_ESM.docx]
